# Can false denials turn fact into fiction? The effect of false denials on memory for self-performed actions

**DOI:** 10.1007/s00426-022-01695-7

**Published:** 2022-06-25

**Authors:** Charlotte A. Bücken, Henry Otgaar, Ivan Mangiulli, Niki Ramakers, Harald Merckelbach

**Affiliations:** 1 Leuven Institute of Criminology, Faculty of Law and Criminology, Catholic University of Leuven, Leuven, Belgium; 2 Forensic Psychology Section, Faculty of Psychology and Neuroscience, Maastricht University, Maastricht, The Netherlands

## Abstract

We examined the mnemonic effects of falsely denying a self-performed action. Specifically, participants (*N* = 30) performed, imagined, or received no instruction about 24 action statements (e.g., “cross your arms”). Next, their memory for whether they had performed, imagined, or did nothing (i.e., received no instructions) with these actions was tested. Subsequently, participants were instructed to repeatedly deny an action they had performed (false denial) and to repeatedly claim to have performed an action they had only imagined (false admission). In a final sorting memory task, 54% (*n* = 16) of participants erroneously indicated, after false admissions, that they had performed the imagined action. None of the participants indicated that they had only imagined an action after false denials, showing that it might be difficult to forget a performed action, even after repeatedly denying it. The current experiment sets the stage for future research to investigate why it seems to be difficult to forget performed actions.

## Introduction

Perpetrators often deny their involvement in a crime. For example, Henning and Holdford (2006) surveyed clinicians who had interviewed 2,824 convicted domestic violence offenders. Denials of details surrounding the crime they had been convicted for were quite common. Indeed, a high level of minimization and denial was present in 63% (*n* = 1,719/ 2,736) of the offenders. Moreover, 54% (*n* = 735/1,350) claimed that the descriptions provided by the victim were untrue. Relatedly, 20–30% of defendants claim amnesia for their crimes (Christianson & Merckelbach, 2004; Mangiulli et al., 2019a). While suspects who engage in denial might be innocent, it is not uncommon that also guilty suspects falsely deny that they committed certain acts to evade or minimize culpability (Otgaar & Baker, 2018).

Recently, research has focused on the mnemonic consequences of false denials showing that such denials have memory undermining effects (e.g., Battista et al., 2021; Bücken et al., 2022; Otgaar et al., 2014; Otgaar et al., 2016a). However, these studies have predominantly examined the effect of false denials on memory from a *passive* perspective (i.e., victims or bystanders; but see also Romeo et al., 2019b). Specifically, participants witnessed some stimuli and were subsequently asked to falsely deny having witnessed several details. In this experiment, we explored the memory effects of false denials from a more *active* perspective (i.e., perpetrator perspective). That is, we tested whether it is possible to forget an action that was initially performed, but then repeatedly denied.

### False denials and memory

Research has recently started to look into the mnemonic effects of false denials. For instance, Otgaar et al. (2016a) instructed undergraduate students to watch pictures or a video, and then participants were either asked to deny in response to each question themselves (i.e., self-induced denial; e.g., “The man did not steal anything”), the experimenter falsely denied in response to the participants’ answer (e.g., when the participant indicated that the man stole something, the experimenter would say “no, the man did not steal anything”), or participants answered honestly (i.e., control group). Self-generated denials led to forgetting of details regarding the interview during which participants lied; an effect dubbed *denial-induced forgetting.* The *denial-induced forgetting* effect has been replicated with different stimuli and memory tests (e.g., Battista et al., 2021; Otgaar et al., 2014, 2016a, 2020, Romeo et al., 2019a). Some studies additionally found that self-generated false denials can undermine memory for the experienced event itself, and not just the interview during which the denial occurred (Battista et al., 2021; Experiment 1 in Otgaar et al., 2020; Romeo et al., 2019a).

An important limitation of this research is that participants were asked to deny details that they witnessed, rather than actions they performed (i.e., self-induced actions). Knowing whether denial-induced forgetting extends to actions is important because the type of actions that perpetrators of crime often deny and claim to have forgotten are actions that they performed themselves- (e.g., stealing a piece of jewellery). A priori, it is unlikely to expect that denial-induced forgetting may extend to self-performed actions. The reason for this is that previous studies have documented that *performing* actions enhances memory compared with conditions in which actions are only passively *witnessed*, presumably because motor information adds to the encoding of the actions (Mohr et al., 1989; Engelkamp & Cohen, 1991; Engelkamp et al., 2004; see also Allen & Waterman, 2015; Hainselin et al., 2017). This effect is also called the *action superiority effect.* The concept of action superiority in memory would suggest that it is difficult to forget self-performed actions after denial, and that perhaps denial-induced forgetting would not apply to memory for actions that were repeatedly denied after their performance.

Beyond action superiority, what would be expected from a theoretical memory perspective concerning the mnemonic effect of false denials on self-performed actions? When considering the mnemonic effects of false denials, it is helpful to relate the execution of a false denial to its antipole: a false admission. When people perform a false admission, they essentially falsely report that they performed an action that was never actually performed (Kassin & Kiechel, 1996). The two could be deemed opposites of each other, and false admissions are much more studied than false denials, providing more theoretical ground. For example, a theory that has been proposed to shed light on the mnemonic effects of false admissions is the source monitoring framework (Johnson & Raye, 1981; Johnson et al., 1993). Source monitoring refers to the process of differentiating between memories from different sources (Johnson & Raye, 1981; Johnson et al., 1993). Both external sources, such as perception, and internal sources (e.g., imagination) can lead to memories. Memories based on an external source usually hold more perceptual and contextual details, whereas memories from an internal source contain more cognitive operations. Source monitoring errors arise when memories from internal sources contain so many perceptual memory characteristics that they are confused with memories from external sources. Indeed, studies showed that by imagining an action repeatedly (Goff & Roediger, 1998; Nichols & Loftus, 2019; Thomas & Loftus, 2002) or by falsely admitting to an action (e.g., Kassin & Kiechel, 1996; Nash & Wade, 2009), participants came to believe that the imagined action was actually carried out.

In line with this, source misattributions have been suggested to be a contributing factor to false reporting after false admissions by different scholars (e.g., Henkel & Coffman, 2004; Schacter, 1999). Certain interrogation tactics can induce false beliefs and false memories in people. Thus, perhaps already a false admission in itself can induce false beliefs and false reports (e.g., Henkel & Coffman, 2004). Indeed, Henkel and Coffman (2004) discussed the idea that in truth-telling contexts a false admission itself (i.e., saying “I did do X”) can indeed induce false belief and false memories that an action was performed even though in reality it was not (Henkel & Coffman, 2004).

At this point we can only speculate about which mechanism drives the mnemonic effects of false admissions. Perhaps, while repeatedly falsely admitting to an action, participants repeatedly think about and imagine that action, thereby strengthening the perceptual characteristics attached to the memory for the action. On the other hand, it is possible that repeated false admissions increase familiarity with the idea that the falsely admitted action was actually performed. Indeed, a concept closely related to source monitoring is *misattributing familiarity* (Jacoby, 1991; Johnson et al., 1993). In highlighting this relation, Johnson et al. (1993) argued that sometimes a familiar concept (i.e., action) is recognised, but due to heuristic judgement processes it can be misattributed to a wrong source. For example, it is possible that if someone repeatedly falsely admits to an action, he or she is less careful in reflecting about the source of their memory when deciding whether an action was performed or not because it is quite familiar to them. Such limited reflection activity may increase source monitoring errors (Jacoby, 1991; Johnson et al., 1993).

When it comes to false denials, it is possible that repeated false denials also lead to repeated retrieval, which could strengthen the perceptual characteristics related to the memory for the action even more, as well as increase familiarity with it. However, due to the action superiority effect (Engelkampp & Cohen, 1991; Engelkamp et al., 2004), this increased familiarity might not induce more source errors. Instead, participants may remember the reason that the action is familiar better, which is that the action was indeed performed. In line with this, research on the relationship between familiarity and source monitoring has found that in some cases, familiarity can indeed also aid correct source monitoring (Mollison & Curran, 2012). Thus, these theories support the idea that increased familiarity through repeated retrieval of false experiences (i.e., as false denials are carried out) may prevent denial-induced forgetting of a performed action. Then, repeatedly falsely denying that an action was performed would not undermine the memory of this act.

### Gaps in research

To the best of our knowledge, only one experiment to date has assessed the effect of false denials on memory from a more involved perpetrator perspective (Romeo et al., 2019b). In this experiment, participants performed a mock crime (i.e., stealing answers to an exam), after which some participants were instructed to falsely deny the crime in a memory interview. Twenty-four hours later, their memory was tested in a source memory task. Romeo et al. (2019b) found that memory for the mock-crime of participants who falsely denied was not impaired, in line with the action superiority effect. A limitation of this research is that the performed action (i.e., the mock crime) was only denied *once.* However, perpetrators of crime could well deny their actions across repeated interviews to give consistent reports (Fisher et al., 2013; Mangiulli et al., 2019b) and no research to date has examined such repeated false denials of performed actions.

A study by Battista et al. (2020) on repeated versus single false denials of a witnessed crime (i.e., theft) found that repeated false denials had even larger memory undermining effects as compared with single false denials of witnessed events. Thus, more research on the mnemonic effects of false denials of self-performed actions is necessary to reproduce the results of Romeo et al. (2019b) using different stimuli, paradigms, and especially repeated false denials. Apart from being theoretically interesting, such research could also bear practical relevance. Specifically, if (repeated) denials may lead to the forgetting of self-performed actions, it could increase our understanding about why some offenders might not fully remember their deeds (i.e., and claim amnesia in legal contexts).

### The present experiment

With these considerations in mind, we examined whether it is possible to forget self-performed actions after falsely denying them. We used a procedure adapted from previous research on self-performed actions and the imagination inflation paradigm (e.g., Cohen, 1981; Goff & Roediger, 1998; Li et al., 2020; Otgaar et al., 2016b; Scoboria et al., 2018; Thomas & Loftus, 2002). Participants performed, imagined, or received no instructions with simple actions (e.g., “cross your arms”, “clap your hands”) that they heard and saw the experimenter perform and then received a memory test. Following this, they had to repeatedly deny or admit that they had performed certain actions and were instructed to lie about some of them by falsely denying one action that they had performed, and falsely admitting to having performed another action that they had only imagined. Finally, participants received another memory test. Thereby, we are extending the limited previous work on false denials of performed actions in two ways. First, we are attempting to replicate findings of Romeo and colleagues (2019b) in a new false denial paradigm, and second, this study is an attempt to extend the findings of single false denials of performed actions to *repeated* false denials of performed actions. From research on false denials from a passive witness perspective, we know that mnemonic effects of denials are stronger if the latter were repeated (Battista et al., 2020), and perpetrators may deny their crimes repeatedly before eventually coming forward (Fisher et al., 2013; Mangiulli et al., 2019b).

We expected that repeatedly falsely denying a performed action would *not* have memory undermining effects for the same action. This is because we expected that repeated false denial leads to repeated retrieval, but does not necessarily reduce the number of perceptual characteristics associated with the memory, in line with source monitoring principles (Johnson & Raye, 1981; Johnson et al., 1993). If false denials did not affect the number of perceptual characteristics associated with the memory, we would not expect participants to make source monitoring mistakes in which they misattribute performed actions to imagined (or done-nothing with) ones after false denial. Consequentially, this is why we did not expect a memory undermining effect of false denial on memory for performed actions. Another reason we did not expect to find such an effect is that repeated false denials may lead to increased familiarity of the denied action and in combination with the action superiority effect (e.g., Engelkamp et al., 2004), this could render it even less likely that false denials would be related to forgetting that the falsely denied action was performed.

In line with previous research on the internalisation of false admissions (e.g., Kassin & Kiechel, 1996; Nash & Wade, 2009), we did expect that falsely admitting non-performed actions repeatedly will alter memory for these imagined actions and lead to false memories. We expect this because false admissions will likely increase memory qualities of the experience and influence familiarity, which can lead to source confusions and misattributions by limiting source reflection at retrieval (e.g., Goff & Roediger, 1998; Jacoby, 1991; Johnson et al., 1993).

## Method

### Participants and design

Thirty undergraduate students took part in the experiment (20 women, 10 men; no participants were excluded; *M*_age_ = 24.9; SD_age_ = 7.99, range = 18–51 years). Using G*Power (Faul et al., 2007), a sensitivity power analysis (*F*-test; ANOVA: Repeated measures, within factor) with an alpha of 0.05, a power of 0.80, and the current sample size (*N* = 30), one group, and six measurements (the three action types across two phases) was performed. This power analysis indicated that with this sample size, the smallest detectable effect is *f* = 0.192 (equivalent to *η*^2^ = 0.035 or *d* = 0.38) for a two by three repeated measures ANOVA comparing memory reports across the two phases and three action types. Participants were required to have sufficient understanding of the Dutch language (i.e., participants were native speakers or followed a university program in the Dutch language). Participants were recruited for a study on “memory for simple actions” through advertisements at Maastricht University and received compensation in the form of course credit or monetary vouchers (5€). A within-subjects design was used in this experiment. The experiment was approved by the standing ethical committee of the Faculty of Psychology and Neuroscience at Maastricht University (Master_188_05_02_2018).

### Materials and procedure

**Fig. 1:**
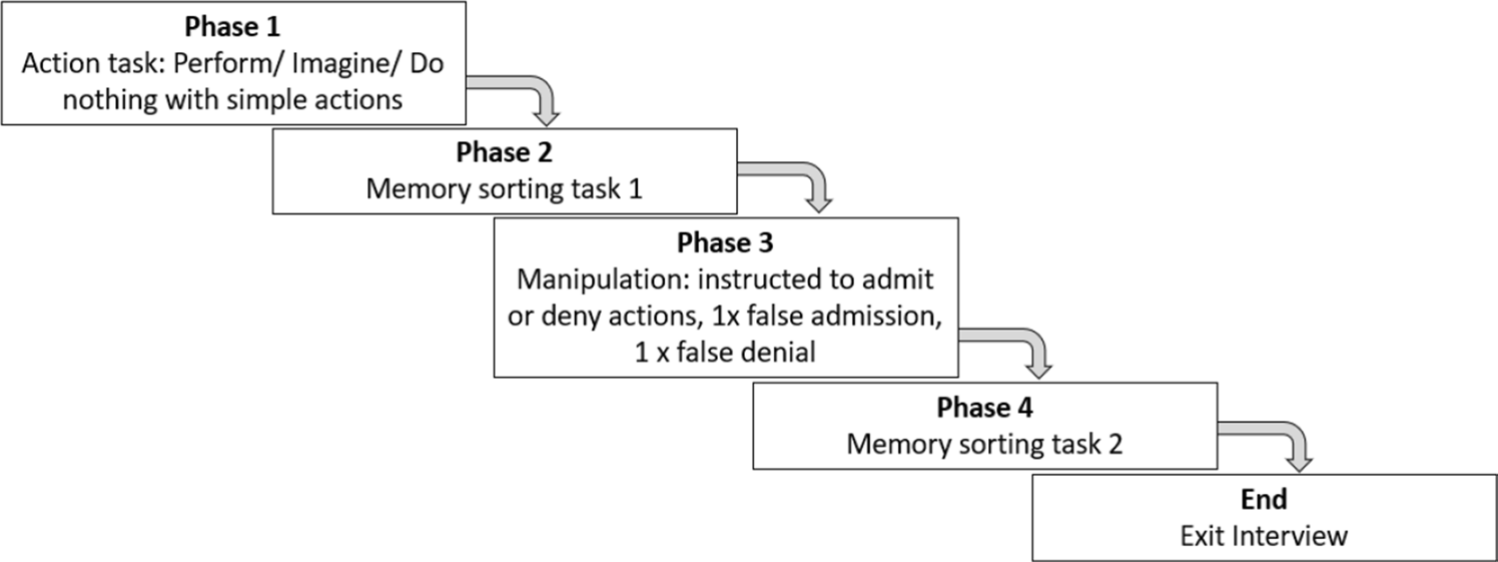
Flow chart of phases within session

All materials and data are available at the Open Science Framework (https://osf.io/gkb85/). The current experiment consisted of four phases that all took place in the same session (approximately half an hour; see Fig. 1). In the first phase, participants signed a written informed consent. Subsequently, the experimenter first read and then performed a series of simple, neutral actions (e.g., “Touch your nose with your left finger”, “Nod Yes”, “Wave your hand”) which were also written on cards shown to participants. Participants were then instructed to either (1) perform (i.e., *“When I ask you to "execute" the action, the intention is that you actually perform the action that is on the card.”*), (2) imagine (i.e., “*When I ask you to "imagine" the action, it is the intention that you only imagine the action on the card. It is important that you really do not perform the action in the instruction "imagine". You can achieve this, for example, by closing your eyes and recalling the action.*”), or (3) do nothing with the actions (i.e., *“When I say "do nothing" then the intention is that you only read the action on the card. The action must therefore not be carried out and must not be imagined.”*). The experimenter monitored closely that the instructions were followed properly (i.e., participants did not perform actions they were told to imagine). In total, we used 24 actions from a standardized set developed by Cohen (1981). The instructions have been used previously in experiments on self-performed tasks (e.g., Goff & Roediger, 1998; Otgaar et al., 2016b; Thomas & Loftus, 2002). The “do nothing” instruction served as a control condition, meaning that participants were neither told to perform nor imagine these actions. Instead, they just heard the experimenter read the action and watched them perform the action (which was done for all action types). In total, participants performed eight actions, imagined eight actions, and received eight actions without instructions (i.e., “do nothing” condition). Blocks of actions were counterbalanced over participants.

In the second phase, participants performed the first memory sorting task (i.e., the baseline memory task). Specifically, participants were instructed to sort all 24 mini action task cards onto three plates; one for “performed” actions, one for “imagined” actions, and one for “do nothing” actions (i.e., actions for which participants received no instructions). All action cards fit into one of these three piles and there were no “foil” cards with actions that were not part of the first phase. On the back of these cards, participants rated their confidence on a visual analogue scale of 0 *not confident at all* to 100 *extremely confident*. This task was self-paced without involvement of the experimenter so as to eliminate any pressure on the participants.

The third phase included the experimental manipulation. First, participants received clear definitions of both admissions (i.e., stating that an action was indeed performed) and denials (i.e., stating that an action was *not* performed). Then, it was explained to them that for each action, a different behaviour was expected from them and to follow specific instructions for each action. After any questions were cleared up, the main task of the third phase started. Specifically, in this task, participants were shown 16 cards with actions (i.e., all “performed” and “imagined” actions from phase 1). Each card came with an instruction telling the participant to say either “I did ….” (i.e., admission) or “I did not…” (i.e., denial) regarding the action shown on the card. For example, participants could receive a card with the action “look at the ceiling” and the instruction “you did this” which would probe them to say “I did look at the ceiling” or the instruction “you did not do this”*,* which would probe them to say “I did not look at the ceiling”. They were instructed to repeat the admissions and denials ten times in succession. In total, there were 160 trials (10 × 16 action statements). For each trial, participants were instructed to perform the admissions and denials in a convincing and believable way, without merely ‘rattling down’ the sentences (i.e., *for each action I will say "you have performed this action" or "you have not performed this action". It is then the intention that you construct the action in a sentence that indicates that you have or have not performed it. Repeat this sentence ten times in a believable way*. *It is important that you follow my instructions and sit still (i.e., not perform the actions in this phase.*). This was meant to ensure that participants would think about their sentences each time and retrieve some perceptual details related to them. For seven of the eight action statements, participants were instructed to (correctly) state ten times that they had performed the action, and for seven of the eight imagination statements participants were instructed to (correctly) state ten times that they had only imagined the action. Further, participants were instructed to deny ten times that they had performed one action (that they had actually performed) and to falsely admit ten times that they performed one action that was only imagined. All admissions and denials were directed at the experimenter (i.e., participants were lying to the experimenter). Actions that were falsely admitted and denied were counterbalanced across participants. Participants had the opportunity to ask for clarification in case they would have trouble understanding what was expected of them.

Finally, in the fourth phase, participants completed the memory sorting task again, following the same procedure as in phase two.^1^ Specifically they received the following instructions: “*[…] So, you are going to put the cards for actions that you performed in the first task on this plate, the actions that you imagined on the “imagine” plate, and the actions you did nothing with at on the plate “not imagined and not performed” […]”.* After completion of the study, participants were asked to complete exit questions. The purpose of these questions was to check participants’ motivation and understanding of the experimental instructions. There were seven questions (e.g., “On a scale of 1–10, how clear were the instructions during this study?”; “On a scale of 1–10, how well did you cooperate during this study?”). Answers were given on a scale of one to ten. One additional open question was included (i.e., “Do you have any remarks regarding the study?”). The results related to the exit interview questions can be found in the supplementary analyses uploaded to the Open Science Framework.

### Scoring

#### Memory sorting task

Participants received one point for each correct sorting in the sorting tasks. Thus, for each of the three categories (i.e., performed actions, imagined actions, and do nothing actions) within both memory tasks (baseline and post-manipulation) sum scores were calculated. The maximal score for each of the three categories was eight. Final scores were proportions, i.e., number of correct sortings divided by eight, leading to six (3 × 2) scores for each participant.

#### Source monitoring errors

Critically, we scored whether participants changed their answer to the falsely denied and falsely admitted critical items from the baseline (phase two) to the post-manipulation memory task (phase four).

## Results

### Baseline memory

On the baseline memory task, participants on average correctly sorted 0.95 of performed actions (7.6/ 8), 0.75 of imagined actions (6/8), and 0.78 (6.2/8) of control actions (Table 1). A within-subjects repeated measures ANOVA indicated a statistically significant effect of category, *F*(2, 58) = 11.23, *p* < 0.001, *η*_*p*_^*2*^ = 0.28. Pairwise comparisons indicated that performed actions were remembered statistically significantly better than both imagined actions, *t* (29) = 5.07, *p* < 0.001, *d* = 0.93, and “nothing” actions, *t* (29) = 4.18, *p* < 0.001, *d* = 0.76. No statistically significant difference between memory for the imagined and “nothing” actions was found, *t* (29) = − 0.47, *p* = 0.64.

**Table 1:** Proportion of participants’ (*N* = 30) correct responses in memory and confidence scores for Phase 2 and Phase 4

Condition	Phase 2				Phase 4			
	Proportion correct		Confidence		Proportion correct		Confidence	
	*M*	*SD*	*M*	*SD*	*M*	*SD*	*M*	SD
Perform	0.95	0.10	9.81	0.34	0.94	0.09	9.7	0.34
Imagine*	0.75	0.22	7.73	1.40	0.67	0.24	7.28	1.95
Nothing	0.78	0.19	7.15	1.86	0.76	0.26	6.82	1.96
Overall*	0.83	0.11	8.23	1.04	0.77	0.12	7.94	1.29

*p < 0.05 between phases, *N* = 30 for all

### True memory

**Fig. 2:**
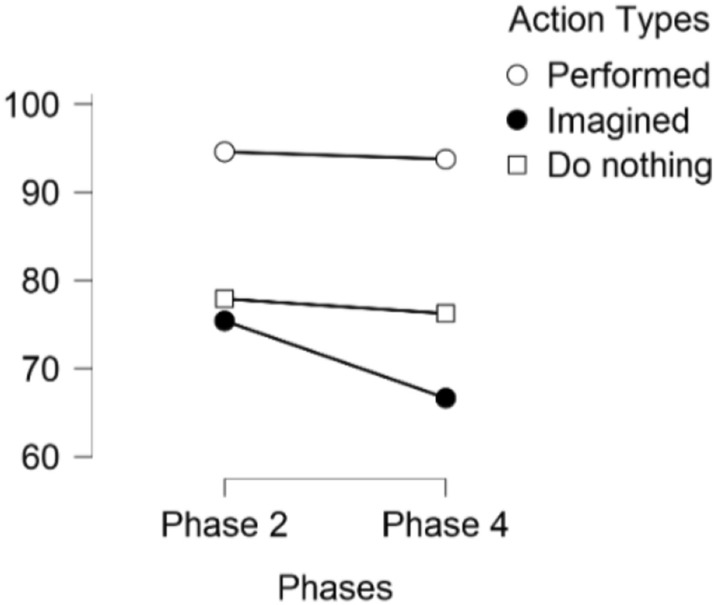
Percentage of correct responses across phases and action types

Table 1 summarizes proportions of correct attributions for the three action conditions and mean confidence ratings during phase four. We performed a 2 × 3 repeated measures ANOVA to compare the proportion of correct responses across the two phases (i.e., phase two and phase four) and the three different types of actions (i.e., imagined, performed, and done nothing with actions). For the two phases, we did not find a statistically significant difference between the percentage of correct classifications in phase two compared with phase four (*F* (1,29) = 2.170, *p* = 0.15, *η*^2^ = 0.01). Looking at correct classifications across the two phases (see Fig. 2) there does seem to be a trend in which imagined actions are classified correctly more often in phase two than phase four, however, this did not reach statistical significance, as also seen in bonferroni post hoc comparisons (*t*(29) = 2.35, *p* = 0.32). We did find a statistically significant difference between correct classifications across the three action types (using Greenhouse–Geisser corrections for violation of the assumption of sphericity: *F* (1.53, 44.25) = 15.1, *p* < 0.001, *η*^2^ = 0.25). Bonferroni post hoc comparisons indicated that performed actions were statistically significantly more often classified correctly than both imagined (*t*(29) = 5.3, *p* < 0.001,* d* = 0.97) and done nothing with actions (*t*(29) = 3.91, *p* < 0.001,* d* = 0.72). Imagined and done nothing with actions did not differ statistically significantly from each other (*t*(29) = -1.38, *p* = 0.52,* d* = 0.25). Lastly, the interaction between the phases and action type was not significant (using Greenhouse–Geisser corrections for violation of the assumption of sphericity: *F* (1.81, 52.22) = 1.71, *p* = 0.19, *η*^2^ = 0.01).

Moreover, we conducted a paired samples t-test to compare confidence scores between phases two and four. Confidence scores did not decline statistically significantly from phase two to phase four, *t*(29) = 1.62, *p* = 0.116,* d* = 0.3. Additional analyses on the confidence scores can be found in the supplementary materials (https://osf.io/xvaf6/).

### Source monitoring errors

**Fig. 3:**
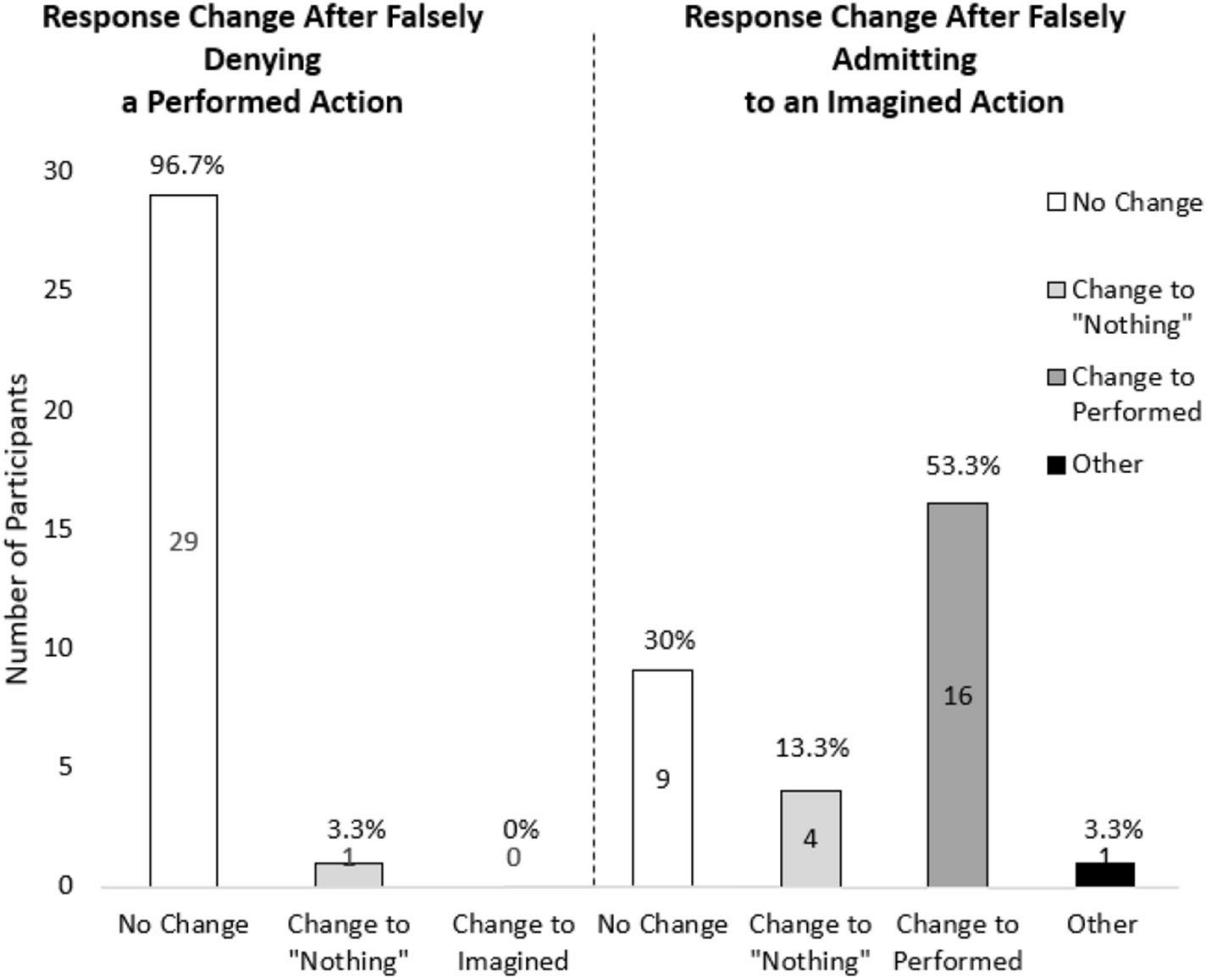
Changes in responses to the memory task (Phase 2 to Phase 4) for the falsely denied and falsely admitted action. Note. “Other” refers to a case in which a participant had falsely sorted the imagined action to the “nothing” pile and then changed it to the “performed” pile after falsely admitting to it

For the falsely denied action, none of the participants made a shift from phase two to four from performed to imagined, meaning that none of the participants thought that they had only imagined a performed action after falsely denying it. However, one participant (3.3%) shifted from performed to “nothing” after falsely denying the action. As for false admissions, 53% (*n* = 16) made a shift from “imagined” to “performed” from phase two to four. Moreover, 13.3% (*n* = 4) changed from “imagined” to “nothing”, and 3.3% (*n* = 1) made another change (see Fig. 3).

## Discussion

We explored whether falsely denying a self-performed action would lead to forgetting performing that action. First, we found that performed actions were generally remembered statistically significantly better than imagined and done-nothing with actions. Second, while a substantial percentage of participants reported having performed an action that they had only imagined in the false admission condition, it was almost impossible for participants to forget an action that they had performed after repeated false denials. It should be noted that the latter finding is a null effect, and that some of our main findings are exploratory in nature. We will now elaborate on the importance of our main findings.

First, we found that performed actions were remembered well, with correct classification scores as high as 94% and 95% in the two memory tests of the current study. Also, we found that memory for performed actions was better than memory for both imagined actions and control actions across both phases. The studies of Engelkamp et al. (2004), Engelkamp & Cohen, 1991 provide a good reason as to the action superiority effect: Performing an act will generate in addition to all the visual cues that are present motor information that helps to strengthen memory traces and make them relatively immune to forgetting and misinformation.

Second—and also in line with the action superiority effect we found—, not a single participant stated that they had imagined a performed action after falsely denying it, although one participant (3.4%) misremembered having only heard about it. Overall, this suggests that in the case of self-performed acts it is nearly impossible to turn fact into fiction and forget that an action was performed even after the person repeatedly denied it. Some previous work on false denials did find an undermining effect of false denials on memory for a witnessed event (i.e., Battista et al., 2021; Otgaar et al., 2020; Romeo et al., 2019a). However, likely because of the action superiority effect (Engelkamp & Cohen, 1991; Engelkamp et al., 2004), no effect of internal false denials on memory for self-performed actions was detected. This result is also in line with the study by Romeo and colleagues (2019b) who did not find an effect of a single false denial on memory for a performed mock crime either.

Moreover, the false admission instructions in the current experiment can be seen as a form of (internal) misinformation. In line with the broader literature on misinformation (for a review see Loftus, 2005), we found that our instructions created false memories (claiming to have performed an act that was only imagined) in a sizeable proportion of participants. Interestingly, this proportion (i.e., 53%) was somewhat higher as compared with that found in false confession studies using the classic alt-key paradigm (e.g., 28% in Kassin & Kiechel, 1996), but smaller than that reported by Nash and Wade (2009) who exposed participants to edited (i.e., in line with the suggestion) video evidence of the action they supposedly committed (63% full internalization).

In sum, we found an asymmetry. That is, it was much easier to induce in people a false memory of an act they did not perform than it was to “erase” a true memory of an act they did perform. This asymmetrical pattern can be explained by drawing on the tenets of the source monitoring framework (Johnson et al., 1993). Specifically, when participants repeatedly falsely admitted to an action, they may have attempted to retrieve imagery of such an action to determine whether they remember having performed it. Even if participants attempted to retrieve imagery only once or twice when falsely admitting to the action, this would have strengthened the perceptual details of this imagery, which ultimately might have led to source monitoring errors (Roediger et al., 2009). Relatedly, it is possible that participants imagined themselves performing the action when they falsely admitted to it repeatedly, leading to increased perceptual details related to the memory and imagination inflation related to source monitoring errors (see e.g., Goff & Roediger, 1998). Lastly, repeated false admissions could have increased familiarity that the action was performed. In turn, such a familiarity could have led to *limited reflective activity* at the retrieval stage and thus an increased likelihood for source monitoring mistakes (Jacoby, 1991; Johnson et al., 1993). Hence, the source monitoring framework is useful to understand how repeated false admissions in the current study led to increased false reporting that this specific imagined action was indeed performed. On the other hand, when participants falsely deny having performed an action, they may paradoxically retrieve its memory repeatedly, thereby strengthening its perceptual characteristics (Roediger et al., 2009; Wegner et al., 1987). Similarly to repeated false admissions, repeated false denials would have led to an increased familiarity with the lied about action. However, combined with the *action superiority* effect, this strengthening of perceptual characteristics and familiarity could have made source monitoring mistakes very unlikely, because participants would be reminded that they in fact did perform the action during false denial.

Some limitations of the current study should be mentioned. First, the sample size of our experiment was rather small (*N* = 30). Yet, considering our sensitivity power analysis, all of the effects we did find in our inferential analyses had effect sizes larger than the effect size that our sensitivity power analysis indicated was the smallest detectable effect size of interest (i.e., > *η*^2^ = 0.035 or *d* = 0.38). Moreover, the most pertinent finding, being that no participants misclassified a performed action as an imagined action after repeatedly falsely denying it, was the result of a frequency analysis (i.e., unrelated to the power analysis). This strongly suggests that *in principle* it might be very unlikely to forget a performed action, even after falsely denying it. Nevertheless, our findings should be replicated using a larger sample. Additionally, to increase participants’ involvement with the repeated admissions and denials (i.e., in phase three) and to prevent participants from merely ‘rattling down’ the repeated lies, future studies might employ a strategy in which the repeated denials and admissions are not told in succession, but in which action statements are intermixed (i.e., and not merely blocked).

Moreover, a limitation pertains to our finding that falsely admitting that an imagined action was performed leads to false reports of actually having performed that action in the final memory phase. A possible confounding factor here is that imagination itself can lead to internalisation that an action was performed, and the formation of false memories and beliefs (e.g., the imagination inflation effect; Cohen, 1981; Goff & Roediger, 1998; Li et al., 2020). Yet we do know from previous research that false admission itself can have the same effect of inducing increased false belief or memory that the event the person falsely admitted to occurred (e.g., Kassin & Kiechel, 1996). Nevertheless, the effects of the false admission itself and of imagining the action cannot fully be separated in our results, because we only asked participants to falsely admit to an imagined, and not also a control (i.e., done nothing with) action. The reasoning for this was that we were predominantly interested in the mnemonic effects of false denials, and wanted to keep the design parsimonious. However, future studies assessing the mnemonic effects of (repeated) false admissions could ask participants to falsely admit to both an imagined and a control (i.e., ‘heard’) action. This would allow for differentiation between the memory effects of the imagination and false admission itself.

Relatedly, we only asked participants to repeatedly falsely deny a performed action, and not falsely deny a ‘heard’ control action. The latter is more akin to (denials of) witnessed actions that a person is not actively involved in, and thus more like the types of actions that have traditionally been studied most in false denial research. Because we were interested in extending the literature on false denials by investigating the mnemonic effects of repeatedly falsely denying *performed* actions, we only asked participants to deny such a performed action. However, it would be interesting in future research if (repeated) false denials of different types of actions were compared directly (i.e., performed actions, witnessed actions, actions one is at the receiving end of). For example, false denials could then be studied and compared using different perspectives (i.e., a perpetrator vs victim vs witness perspective).

An additional point of consideration for future studies examining the memory consequences of false denials of performed actions is that, we only tested memory immediately after the false denial, within the same session. At that time point, false denials did not seem to impact memory of performed actions, perhaps due to the action superiority effect and because performed actions are remembered very well. However, it is possible that after a delay (e.g., 48 h or a week), false denials would undermine memory for a performed action. This might depend on the robustness and boundary conditions of the action superiority effect. Although not always, some other research on false denials has sometimes included a delay period after the denial (e.g., Battista et al., 2020; Otgaar et al., 2014), and research on performed actions in the imagination inflation paradigm has typically included a longer delay period as well (e.g., Goff & Roediger, 1998; Thomas & Loftus, 2002). Thus, future research could employ another memory test after a longer delay (e.g., 24 h, 48 h, or a week) after participants falsely deny a performed action.

Another limitation is that participants in the current study were university students, who arguably are different in many respects from perpetrators who come to falsely deny their violent acts in court. Also, the current study only looked at memory for simple, neutral actions. While past studies on the mnemonic effects of false denials have not always found an effect of denials on memory for the target event (i.e., most often memory for the time at which the lie occurred is impaired), some that did find such an effect used highly negatively emotional stimuli (Romeo et al., 2019a; for a summary, see Otgaar & Baker, 2018). Thus, future studies could examine whether it is possible that internal false denials undermine memory for complex, emotionally negative actions.

Because of these limitations and the exploratory nature of some of our results, there is a need for further research to replicate the results of our study in a larger sample. That way, future research could also determine the reasons that it might be difficult to forget performed actions, even after false denial. Generally, such future studies could address limitations of the current experiment by (1) intermixing the repeated denials or admissions of the actions, (2) creating an additional control condition in which not just an imagined, but also a heard action is falsely denied, (3) testing memory for the performed actions after a delay, and 4) testing the effects of falsely denying different valenced actions.

## Conclusions

We found that repeatedly denying an action that was performed does not lead to forgetting that action. Admittedly, absence of evidence is not evidence of absence. There is the remote possibility that developing amnesia for outstanding acts due to denial, suppression, blocking or dissociation does exist. But to claim that this scenario must be true because it has not yet been proven false would be an argument from ignorance.

## Data Availability

The datasets analysed during the current study are available in the Open Science Framework repository (https://osf.io/gkb85/).

## Abbreviations

DES: Dissociative Experiences Scale
TAFQ-A: Thought Action Fusion Questionnaire-Adolescent Version

## Footnotes

1 As an exploratory corollary, two individual difference questionnaires, one about dissociative symptoms and the other about though-action fusion (i.e., the DES and TAFQ-A), were administered. These data were analyzed and can be found in the supplementary material uploaded to the Open Science Framework (https://osf.io/e3mqt/).

## Declarations

### Open practices statement

All raw data and materials are available at the Open Science Framework (https://osf.io/gkb85/). The experiment was not preregistered.
